# Evaluation of the impact of the state of emergency during the COVID-19 pandemic on childhood immunizations in Benguela Province, Angola

**DOI:** 10.1186/s41182-024-00668-3

**Published:** 2025-01-14

**Authors:** Tadatsugu Imamura, Keiji Mochida, Balogun Olukunmi, Lino Tchicondingosse, Pedro Sapalalo, Ketha Rubuz Francisco, Ai Aoki, Akira Ishiguro, Kenji Takehara

**Affiliations:** 1 Center for Postgraduate Training and Education, National Center for Child Health and Development, 2-10-1 Okura, Setagaya-Ku, Tokyo, 〒157-8535 Japan; 2 Department of Health Policy, National Center for Child Health and Development, Tokyo, Japan; 3 Domus Custodius (SU) Lda, Tchikos Agency, Luanda, Angola; 4 National Directorate of Public Health, Ministry of Health, Luanda, Angola

**Keywords:** COVID-19, Pandemics, Immunization programs, Cohort studies, Angola

## Abstract

**Background:**

The impact of public health measures against the coronavirus disease 2019 on the rate of childhood immunization has not yet been fully defined. Particularly, measures which directly affect health-seeking behaviors (e.g., the State of Emergency; SoE) drew public health attention. We aim to describe this impact in Benguela Province, Angola, by comparing the immunization rates between infants who had their immunizations before and after the SoE, which was declared on March 27, 2020.

**Methods:**

We retrospectively reviewed the epidemiological data of infants born between November 2019 and February 2020 in Benguela Province, Angola. Immunization rates (i.e., the number of immunized infants divided by the number of infants in the group of same months of birth and residential areas) were calculated for 11 vaccines that infants received from at birth to the 4th month after birth. The rates for the 2nd month vaccines were compared between infants immunized before the SoE (post-SoE), and after the SoE (pre-SoE).

**Results:**

Among 9,595 infants, the overall immunization rates were higher in the post-SoE (71.9–77.8%) than in the pre-SoE groups (66.0–73.8%). The overall immunization rates were higher in the post-SoE group than in the pre-SoE group in both urban and rural municipalities, although the rates were > 20% higher in urban than in rural municipalities. The immunization rates in the recommended month showed a similar trend, except for the stratified analysis for rural municipalities, where the rates were 2.3–4.1% lower in the post-SoE than in the pre-SoE groups.

The most common reason for missing immunization was vaccine unavailability at health units (19.9%, 684/3,440). Less than 10% of missed immunizations were due to the SoE, which occurred mostly in infants born in rural municipalities in February 2020 (9.8%, 52/532). Less than 2% of missed immunizations were due to health units not being open, and was highest in rural municipalities in January 2020 (1.6%, 27/1,673).

**Conclusions:**

Our study suggested that the disruptive impacts of public health measures against pandemics on rates of childhood immunization can be mitigated, and support is needed for areas with vulnerable health systems, such as rural areas.

**Supplementary Information:**

The online version contains supplementary material available at 10.1186/s41182-024-00668-3.

## Introduction

The coronavirus 2019 (COVID-19) pandemic has disrupted various health care services, including routine childhood immunizations worldwide [1–3]. In Africa, COVID-19 pandemic was reported to be attributable for the delayed introduction of rotavirus vaccine and a significant decrease of immunization coverage for measles vaccine [4, 5]. In the effort to elucidate the underlying mechanisms for such global trend, the impact of public health measures, particularly those directly affect people’s mobility and health-seeking behaviors (e.g., the state of emergency; SoE), have drawn public health attention [6–8]. It is currently unknown whether such impacts were mitigated in countries with policies to continue their routine health care services, as the strictness of public health measures varied between countries [9]. Lack of scientific evidence in this topic hinders public health authorities worldwide from formulating effective immunization programs during epidemics. Studies on the effects of public health measures on childhood immunizations in low-income countries, particularly sub-Saharan African countries, are very scarce.

We aim to describe the impact of the public health measures against the COVID-19 pandemic, particularly the SoE, on the childhood immunization program in a provincial area of Angola.

## Materials and methods

### Study design

This study is a secondary data analysis using data from our previous randomized control trial (RCT) in Benguela Province, Angola, which aimed to determine the impact of the Maternal and Child Health handbook (MCHHb) on public health services [10, 11]. We randomly allocated all 10 municipalities in Benguela Province into either the intervention or control group. All public health care facilities providing MNCH services located in Benguela Province were included. All women pregnant at the beginning of the trial period and those who visited a health facility for antenatal checkups, postnatal checkups, or delivery care were eligible for including in the study. Out of 11,530 women who were approached, 9,039 women were included into the final analysis of RCT [11].

We retrospectively reviewed the epidemiological data of mothers of 9,595 infants, including 898 born in November 2019, 3,655 in December 2019, 3,697 in January 2020, and 1,345 in February 2020, which was collected during our previous RCT [10, 11]. Participants of the RCT had structured interviews twice: at the enrollment to collect baseline information and at the 3-month postpartum follow-up to collect additional information, including months and places of delivery, months (not dates) and types of immunizations of their children, and reasons for missing immunizations. Immunization records were collected from the MCHHb. Information was validated and registered in the project database for analysis. The ethical considerations of the RCT are detailed in our previous articles [11, 12].

### Immunization rates

Infants were categorized into four groups based on month of birth, then further stratified based on residence in urban or rural municipalities, following the definition in the Demographic Health Survey 2015–16 [13]. Immunization rates (%) were calculated by two approaches: (1) by dividing the number of immunized infants (those who had immunization records regardless of the time) by the number of infants in the group (the overall immunization rates), and (2) by dividing the number of immunized infants in the recommended months of immunizations, by the number of infants in the group.

### Impact of the state of emergency

In Angola, the SoE was declared on March 27, 2020 [14]. Immunizations that were carried out in and after April 2020 were regarded as being under the impact of the SoE (post-SoE), and immunizations in and before February 2020 were regarded as being free from the impact (pre-SoE) Immunization rates were compared between the pre- and post-SoE groups for each of the 2nd month immunizations.

### Statistical analysis

Statistical analysis was carried out using R ver.3.5.0 (R Foundation for Statistical Computing, Vienna, Austria) without imputation for missing data.

## Results

### Immunization rates in Benguela Province

**Fig. 1:**
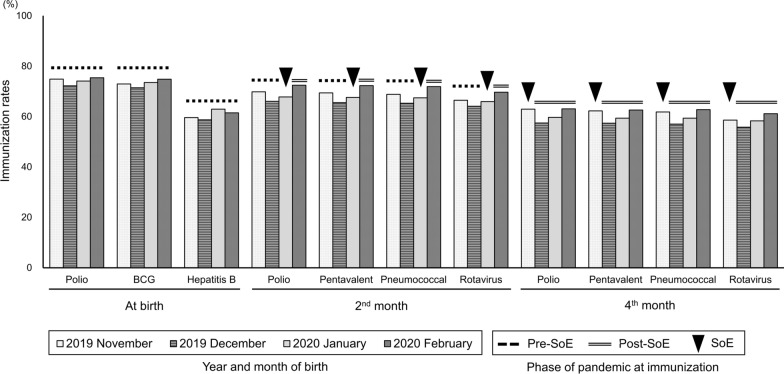
Immunization rates among Angolan infants born between November, 2019 and February, 2020. Bars indicate the immunization rates (%) of the 11 childhood immunizations in Benguela Province, Angola. Markers above bars indicate the phase of the COVID-19 pandemic at the timing of each immunization

The immunization rates ranged from 56% (the 2nd dose of rotavirus vaccine of infants born in December 2019) to 75% (the zero dose of polio vaccine of infants born in November 2019 and February 2020, and the single dose of Bacille Calmette–Guerin (BCG) of infants born in February 2020) (Fig. 1).

### Impact of the SoE on immunization rates

The overall immunization rates for the 2nd month immunizations were higher in the post-SoE than in the pre-SoE group for all four vaccines (Fig. 1, Table 1). The overall immunization rates were also higher in the post-SoE than in the pre-SoE group in urban and rural municipalities, although the rates were > 20% higher in urban than in rural municipalities (Table 1). The total immunization rates in the recommended months (i.e., 2nd month after birth) did not differ significantly between the pre- and post-SoE groups (Table 1). For the 1st dose of the pentavalent vaccine, the immunization rates in the recommended month ranged from 61.4 to 65.9% and 36.1 to 42.4% in urban and rural municipalities, respectively (Table 2). In rural municipalities, the immunization rates in the recommended month were lower in the post-SoE group than in the pre-SoE group by 2.3–4.1% (Table 2). Twelve percent of infants in the post-SoE group (i.e., those born in February 2020) and 6.7–8.4% in the pre-SoE group had their catch-up immunizations in the month after the recommended month (Table 2). Similar results were found for other 2nd month immunizations.

**Table 1:** The immunization rates for the 2nd month’s immunizations of the pre- and post-SoE groups in Benguela Province

	Total		Urban municipalities		Rural municipalities	
	Pre	Post	Pre	Post	Pre	Post
Overall immunization rates						
Polio	3,361 (73.8)	1,046 (77.8)	1,754 (79.0)	604 (83.0)	1,175 (54.9)	330 (58.7)
Pentavalent	3,024 (66.4)	972 (72.3)	1,748 (78.7)	601 (82.6)	1,159 (54.2)	331 (58.9)
Pneumococcal	3,007 (66.0)	967 (71.9)	1,736 (78.2)	599 (82.3)	1,157 (54.1)	329 (58.5)
Rotavirus	3,309 (72.7)	1.045 (77.7)	1,702 (76.6)	585 (80.4)	1,125 (52.6)	316 (56.2)
Immunization rates in the recommended months						
Polio	2,338 (51.4)	699 (52.0)	1,389 (62.5)	468 (64.3)	860 (40.2)	201 (35.8)
Pentavalent	2,338 (51.4)	698 (51.9)	1,397 (62.9)	465 (63.9)	853 (39.9)	203 (36.1)
Pneumococcal	2,329 (51.2)	694 (51.6)	1,389 (62.5)	464 (63.7)	853 (39.9)	201 (35.8)
Rotavirus	2,304 (50.6)	686 (51.0)	1,371 (61.7)	457 (62.8)	847 (39.6)	200 (35.6)

Immunization rates [number (%)] were calculated for the 2nd month immunizations

**Table 2:** Timeliness of immunizations for the 1st dose of pentavalent vaccines in Benguela Province

Area	Month of birth		Total births [*n* (%)]	Month of immunization [*n* (%)]									Total immunized [*n* (%)]
				2019		2020							
				Nov	Dec	Jan	Feb	Mar	Apr	May	Jun	Jul	
Urban	2019	Nov	477 (100)	1 (0.2)	11 (2.3)	**293 (61.4)**	49 (10.3)	24 (5.0)	7 (1.5)	6 (1.3)	2 (0.4)	2 (0.4)	396 (83.0)
		Dec	1744 (100)	0 (0.0)	8 (0.5)	34 (1.9)	**1104 (63.3)**	133 (7.6)	34 (1.9)	16 (0.9)	10 (0.6)	6 (0.3)	1346 (77.2)
	2020	Jan	1788 (100)	0 (0.0)	0 (0.0)	11 (0.6)	41 (2.3)	**1179 (65.9)**	140 (7.8)	35 (2.0)	16 (0.9)	12 (0.7)	1438 (80.4)
		Feb	728 (100)	0 (0.0)	0 (0.0)	2 (0.3)	5 (0.7)	39 (4.1)	**465 (63.9)**	62 (8.5)	28 (3.8)	5 (0.7)	601 (82.6)
Rural	2019	Nov	393 (100)	3 (0.8)	4 (1.0)	**151 (38.4)**	33 (8.4)	12 (3.1)	4 (1.0)	3 (0.8)	0 (0.0)	2 (0.5)	214 (54.5)
		Dec	1746 (100)	0 (0.0)	9 (0.5)	23 (1.3)	**702 (40.2)**	117 (6.7)	46 (2.6)	25 (1.4)	10 (0.6)	10 (0.6)	944 (54.1)
	2020	Jan	1770 (100)	0 (0.0)	2 (0.1)	11 (0.6)	39 (2.2)	**751 (42.4)**	90 (5.1)	45 (2.5)	19 (1.1)	13 (0.7)	976 (55.1)
		Feb	562 (100)	0 (0.0)	1 (0.2)	0 (0.0)	4 (0.7)	17 (3.0)	**203 (36.1)**	69 (12.3)	22 (3.9)	4 (0.7)	331 (58.9)

Immunization rates [number (%)] were calculated for each month between November 2019 and July 2020, and in total. Immunization rates [number (%)] in the recommended month of immunizations (e.g., 2nd month after birth) are highlighted in bold fonts

### Reasons for missing immunizations

**Fig. 2:**
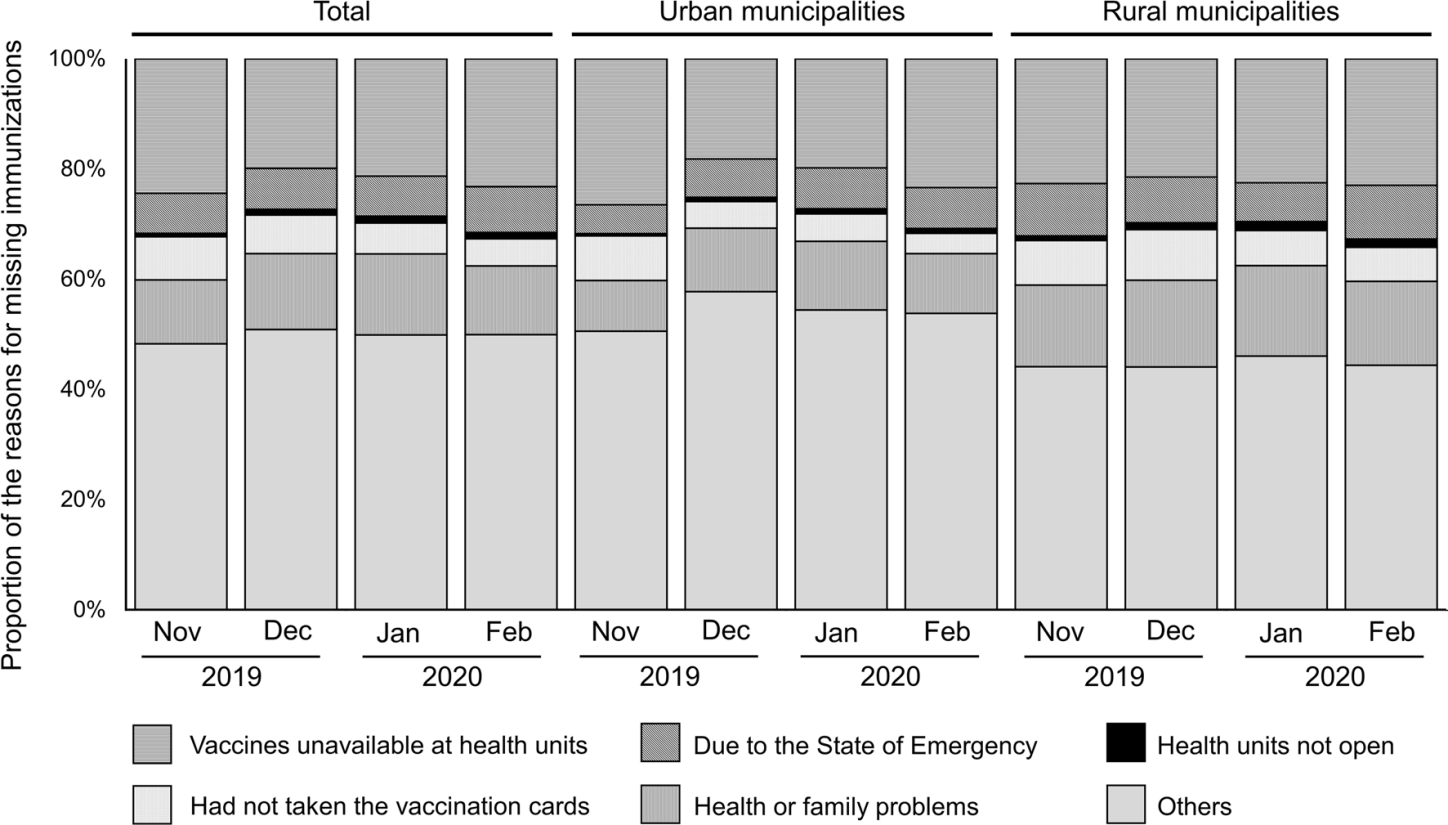
Reasons for missing immunization among Angolan infants born between November, 2019 and February, 2020. Bars indicate the proportion (%) of the reasons for missing immunizations in Benguela Province, Angola

A total of 50.1% (4,639/9,252) of the study participants reported that they missed at least one immunization during the study period. Among the reasons for missed immunization, vaccine unavailability at health units accounted for from 18.2% (298/1,640, December 2019) to 26.5% (121/457, November 2019) in urban municipalities, and from 21.5% (353/1,644, December 2019) to 22.9% (122/532, February 2020) in rural municipalities (Fig. 2). Less than 10% of missed immunizations were due to the SoE, which occurred mostly in rural municipalities in February 2020 (9.8%, 52/532). Less than 2% of missed immunizations were due to health units not being open, and was highest in rural municipalities in January 2020 (1.6%, 27/1,673) (Fig. 2).

## Discussion

The overall immunization rates of the 2nd month immunizations were not significantly reduced under the impact of the SoE in Benguela Province, Angola. Although previous reports have described a reduced coverage of childhood immunizations during the pandemic in low- and middle-income countries, such trends were not found in our cohort [2, 4, 7, 8].

Our results might suggest the resilience of the childhood immunization program in Benguela Province in the face of epidemics. During the early phase of the pandemic, the Angolan government made a policy to maintain routine health care services [14]. In our study, missed immunizations due to a closure of health units were scarce. Continuation of routine health care services might have been helpful in preventing the disruption of childhood immunizations. Our results were in line with a report from Kenya, which described minimal impacts of the pandemic on the childhood immunization coverage [15]. In Kenya, multiple contingency measures were in place, including a separation of immunization services and COVID-19 centers, and a distribution of extra vaccines to provincial health offices before the pandemic [15, 16]. This implies the importance of preemptive contingency plans before the surge of cases to maintain the childhood immunization programs during epidemics.

However, we found decreasing immunization rates in the recommended months in rural municipalities. This might have been due to sociomedical factors, including limited access to health care services and health-related knowledge, and a reduced distribution of vaccines [17, 18]. In fact, unavailability of vaccines was the biggest reason for missed immunizations in our study. Immunization programs during public health crisis might benefit from first focusing on support for vulnerable areas (e.g., rural areas). In our study, the immunization rates in the recommended month, but the overall rates, were reduced in rural municipalities. This indicated that infants had access to catch-up immunizations. Our results suggested that enhanced catch-up immunization programs are warranted to mitigate the negative impacts of such public health crisis in those areas [19, 20].

The limitations of our study include a lack of information about the health-seeking behaviors and other immunization-related interventions, a short study period < 1 year, and the effects of the seasonality (e.g., rainy season) [21–23]. A longer period of observation might be useful in determining their associations. A potential sampling bias cannot be ruled out, as there might have been missing data in the immunization records of MCHHb. However, this was a small proportion and was not associated with any specific areas or months.

We used a dataset which includes both control and intervention groups of the RCT. Although it was assumed to have a minimal effect on our analysis, the immunization rates in the recommended months were higher in the intervention than the control groups (Supplementary Tables 1–2). This might suggest the impact of MCHHb on facilitating timely immunizations, as previous reported [24].

## Conclusions

The childhood immunization rates were not significantly reduced under the impact of the SoE in Benguela Province, Angola. With effective contingency measures to continue routine health care services, disruptive impacts of the childhood immunizations could be mitigated during a public health crisis. Health authorities should focus on supporting immunization programs and vaccine distributions, especially in areas with vulnerable health systems.

## Supplementary Information

Supplementary material 1.

## Data Availability

The datasets used and/or analyzed during the current study are available from the corresponding author on reasonable request.

## Abbreviations

COVID-19: Coronavirus disease 2019
SoE: State of emergency
RCT: Randomized control trial
MCHHb: Maternal and Child Health handbook
BCG: Bacille Calmette–Guerin
