# Supplementary material for: Evaluation of the impact of the state of emergency during the COVID-19 pandemic on childhood immunizations in Benguela Province, Angola
Source: Trop Med Health. 2025 Jan 14;53:5. doi: 10.1186/s41182-024-00668-3 (PMC11730145; doi:10.1186/s41182-024-00668-3)
Supplement: Supplementary file 1 — Supplementary material 1. [file 41182_2024_668_MOESM1_ESM.docx]

Supplementary table 1. The immunization rates for the 2^nd^ months immunizations of the pre- and post-SoE groups in intervention and control groups, Benguela province

|  | Intervention group | | Control group | |
| --- | --- | --- | --- | --- |
|  | Pre | Post | Pre | Post |
| Overall immunization rates | | | | |
| Polio | 1,058  (56.2) | 349  (62.1) | 1,988  (74.4) | 625  (79.8) |
| Pentavalent | 1,053  (56.0) | 349  (62.1) | 1,971  (73.8) | 623  (79.6) |
| Pneumococcal | 1,050  (55.8) | 348  (61.9) | 1,957  (73.2) | 619  (79.1) |
| Rotavirus | 1,025  (54.5) | 338  (60.1) | 1,916  (71.7) | 599  (76.5) |
| Immunization rates in the recommended months | | | | |
| Polio | 1,607  (60.2) | 499  (63.7) | 731  (38.8) | 200  (35.6) |
| Pentavalent | 1,607  (60.2) | 498  (63.6) | 731  (38.8) | 200  (35.6) |
| Pneumococcal | 1,598  (59.8) | 496  (63.3) | 731  (38.8) | 198  (35.2) |
| Rotavirus | 1,576  (59.0) | 490  (62.6) | 728  (38.7) | 196  (34.9) |

Immunization rates [number (%)] were calculated for the 2^nd^ month immunizations.

Supplementary table 2. Timeliness of immunizations for the 1^st^ dose of pentavalent vaccines in intervention and control groups, Benguela province

| Group | Area | Month  of birth | | Total births  [n (%)] | Month of immunization [n (%)] | | | | | | | | | Total immunized  [n (%)] |
| --- | --- | --- | --- | --- | --- | --- | --- | --- | --- | --- | --- | --- | --- | --- |
|  |  |  |  |  | 2019 | | 2020 | | | | | | |  |
|  |  |  |  |  | Nov | Dec | Jan | Feb | Mar | Apr | May | Jun | Jul |  |
| Intervention | Urban | 2019 | Nov | 128  (100) | 1  (0.8) | 4  (3.1) | **60**  **(46.9)** | 18  (14.1) | 12  (9.4) | 4  (3.1) | 2  (1.6) | 0  (0) | 0  (0) | 101  (78.9) |
|  |  |  | Dec | 460  (100) | 0  (0) | 1  (0.2) | 14  (3.0) | **241**  **(52.4)** | 49  (10.7) | 15  (3.3) | 7  (1.5) | 5  (1.1) | 2  (0.4) | 334  (72.6) |
|  |  | 2020 | Jan | 529  (100) | 0  (0) | 0  (0) | 2  (0.4) | 22  (4.2) | **283**  **(53.5)** | 51  (9.6) | 12  (2.3) | 6  (1.1) | 3  (0.6) | 379  (71.6) |
|  |  |  | Feb | 221  (100) | 0  (0) | 0  (0) | 0  (0) | 1  (0.5) | 13  (5.9) | **111**  **(50.2)** | 24  (10.9) | 12  (5.4) | 2  (0.9) | 163  (73.8) |
|  | Rural | 2019 | Nov | 234  (100) | 2  (0.9) | 3  (1.3) | **84**  **(35.9)** | 22  (9.4) | 6  (2.6) | 2  (0.9) | 1  (0.4) | 0  (0) | 1  (0.4) | 121  (51.7) |
|  |  |  | Dec | 977  (100) | 0  (0) | 6  (0.6) | 9  (0.9) | **319**  **(32.7)** | 67  (6.9) | 22  (2.3) | 13  (1.3) | 8  (0.8) | 6  (0.6) | 450  (46.1) |
|  |  | 2020 | Jan | 943  (100) | 0  (0) | 2  (0.2) | 4  (0.4) | 16  (1.7) | **308**  **(32.7)** | 52  (5.5) | 24  (2.5) | 13  (1.4) | 11  (1.2) | 430  (45.6) |
|  |  |  | Feb | 326  (100) | 0  (0) | 1  (0.3) | 0  (0) | 2  (0.6) | 12  (3.7) | **85**  **(26.1)** | 54  (16.6) | 13  (4.0) | 2  (0.6) | 169  (46.7) |
| Control | Urban | 2019 | Nov | 349  (100) | 0  (0) | 7  (2.0) | **233**  **(66.8)** | 31  (8.9) | 12  (3.4) | 3  (0.9) | 4  (1.1) | 2  (0.6) | 2  (0.6) | 294  (84.2) |
|  |  |  | Dec | 1,284 (100) | 0  (0) | 7  (0.5) | 20  (1.6) | **863**  **(67.2)** | 84  (6.5) | 19  (1.5) | 9  (0.7) | 5  (0.4) | 4  (0.3) | 1,011  (78.7) |
|  |  | 2020 | Jan | 1,259 (100) | 0  (0) | 0  (0) | 9  (0.7) | 19  (1.5) | **896**  **(71.2)** | 89  (7.1) | 23  (1.8) | 10  (0.8) | 9  (0.7) | 1,055  (82.2) |
|  |  |  | Feb | 507  (100) | 0  (0) | 0  (0) | 2  (0.4) | 4  (0.8) | 17  (3.4) | **354**  **(69.8)** | 38  (7.5) | 16  (3.2) | 3  (0.6) | 434  (85.6) |
|  | Rural | 2019 | Nov | 159  (100) | 0  (0) | 1  (5.3) | **67**  **(42.1)** | 11  (6.9) | 6  (3.8) | 2  (1.3) | 2  (1.3) | 0  (0) | 1  (0.6) | 91  (57.2) |
|  |  |  | Dec | 769  (100) | 0  (0) | 3  (0.4) | 14  (1.8) | **383**  **(49.8)** | 50  (6.5) | 24  (3.1) | 12  (1.6) | 2  (0.3) | 4  (0.5) | 492  (64.0) |
|  |  | 2020 | Jan | 827  (100) | 0  (0) | 0  (0) | 7  (0.8) | 23  (2.8) | **443**  **(53.6)** | 38  (4.6) | 21  (2.5) | 6  (0.7) | 2  (0.2) | 540  (65.3) |
|  |  |  | Feb | 236  (100) | 0  (0) | 0  (0) | 0  (0) | 2  (0.8) | 5  (2.1) | **118**  **(50.0)** | 15  (6.4) | 9  (3.8) | 2  (0.8) | 151  (64.0) |

Immunization rates [number (%)] were calculated for each month between November 2019 and July 2020, and in total. Immunization rates [number (%)] in the recommended month of immunizations (e.g., 2^nd^ month after birth) are highlighted in bold fonts.
